# Maintaining bovine satellite cells stemness through p38 pathway

**DOI:** 10.1038/s41598-018-28746-7

**Published:** 2018-07-17

**Authors:** Shijie Ding, G. N. M Swennen, Tobias Messmer, Mick Gagliardi, Daniël G. M. Molin, Chunbao Li, Guanghong Zhou, Mark J. Post

**Affiliations:** 1 Department of Physiology, Maastricht University, Maastricht, 6229 ER The Netherlands; 2 Key Lab of Meat Processing and Quality Control, College of Food Science and Technology, Nanjing Agricultural University, Nanjing, 210095 China

## Abstract

Isolating and maintaining the appropriate stem cell for large scale cell culture is essential in tissue engineering or food production. For bovine satellite cells an optimized isolation and purification protocol is lacking and there is also no detailed understanding on the factors that maintain stemness of these cells. Here, we set up a fluorescence-activated cell sorting strategy to enrich bovine satellite cells. We found that p38-MAPK signalling is activated and PAX7 expression is gradually lost during satellite cell proliferation. The p38 inhibitor (SB203580) treatment maintained PAX7 expression but inhibited the fusion of satellite cells in a concentration-dependent way in short-term incubation. The mechanism of p38 inhibition was confirmed by inhibiting canonical p38 signalling, i.e. HSP27. Long-term culture with an appropriate concentration of p38i enhanced the proliferation and PAX7 expression, while the differentiation capacity recovered and was enhanced compared to vehicle control. These studies indicate that bovine satellite cells maintenance depends on cell purity and p38 MAPK signalling. Inhibition of p38 MAPK signaling is a promising strategy to facilitate large scale cell expansion of primary cells for tissue engineering and cultured meat purposes.

## Introduction

Satellite cells, initially identified by Mauro^1^ in 1961, are the bona fide muscle stem cells. These cells are located beneath the sarcolemma and the basal membrane and originate from the dermomyotome cell population^2^. During peri- and postnatal development, satellite cells contribute new nuclei to growing muscle fibers by fusing with the adjacent fiber^3,4^. Subsequently, they enter a quiescent stage and are activated in injured muscle or for further muscle growth^3,5,6^. Understanding the biology of satellite cells will help understand skeletal muscle regeneration, ageing, disease^5^ as well as the emerging field of culturing meat. Culturing meat for consumption uses stem cells to culture muscle tissue for future meat consumption with potential benefits for the environment, animal welfare and food security^7,8^. This technology depends heavily on the ability of satellite cells to expand to high numbers of cells, for instance by maintaining their stemness while providing fast-growing myoblast colonies^7,9^.

Maintaining or improving stemness, requires a highly purified satellite cell population. Bovine satellite cells are usually isolated by the preplating method^10,11^, which in the absence of further purification leads to a purity of 31% based on fusion index or 95% by DESMIN staining^11,12^. Satellite cells can be further purified using cell surface markers, but these are mostly characterized for mice and humans^13–15^, not for cattle. Highly purified mouse^16^, human^15,17^ and pig^18^ satellite cell populations can be obtained by fluorescence-activated cell sorting (FACS) using CD34, β7-integrin, CD56 or CD29^15,16,18,19^. Expression of these markers is species specific. For instance, mouse satellite cells express CD34, whereas human satellite cells do not^16,17,20^. Bovine satellite cell marker expression is not well characterized. We therefore first set out to purify the initial population of bovine satellite cells, based on marker expression.

Further maintenance of satellite cell stemness can depend on cell signaling during proliferation. p38, a subgroup of the MAPKs, can be activated by stress signals, inflammatory cytokines, and many other stimuli and has been implicated in cell proliferation, senescence, apoptosis and other cellular processes^21,22^. The p38α/β MAPK signaling pathway regulates asymmetric division of satellite cells^23^. One daughter cell activates p38α/β MAPK, induces MyoD expression and generates a proliferating myoblast^23^. In the other daughter cell p38α/β MAPK signaling is not activated and MyoD is not induced, thus renewing the quiescent satellite cell to maintain the stem cell pool^23^.

Previous studies have noted that p38-MAPK signaling plays an important role in the loss of stemness in satellite cells^17,22,24,25^. Acute injury in p38α-deficient mice resulted in a prolonged satellite cell response and an increased stem cell pool^25^. Conversely, elevated activity of p38α/β MAPK signaling induced regenerative defects in older satellite cells compared with younger ones^24^. In the same model, inhibition of p38α/β MAPK signaling and culture on soft hydrogel substrates rejuvenated older satellite cells’ potential for regeneration^24^.

In a purified bovine satellite cell population, we investigated if bovine satellite cells showed up-regulated p38 MAPK signaling accompanied by a loss of differentiation ability during long-term culturing *in vitro* and if p38 inhibition can rescue stemness of satellite cells. Specifically, we found that the p38α/β inhibitor SB203580 inhibited the differentiation of bovine satellite cells in short-term experiments while long-term cultivation with p38i helped maintain the stemness and differentiation abilities.

## Results

### FACS purification of bovine satellite cells

**Figure 1:**
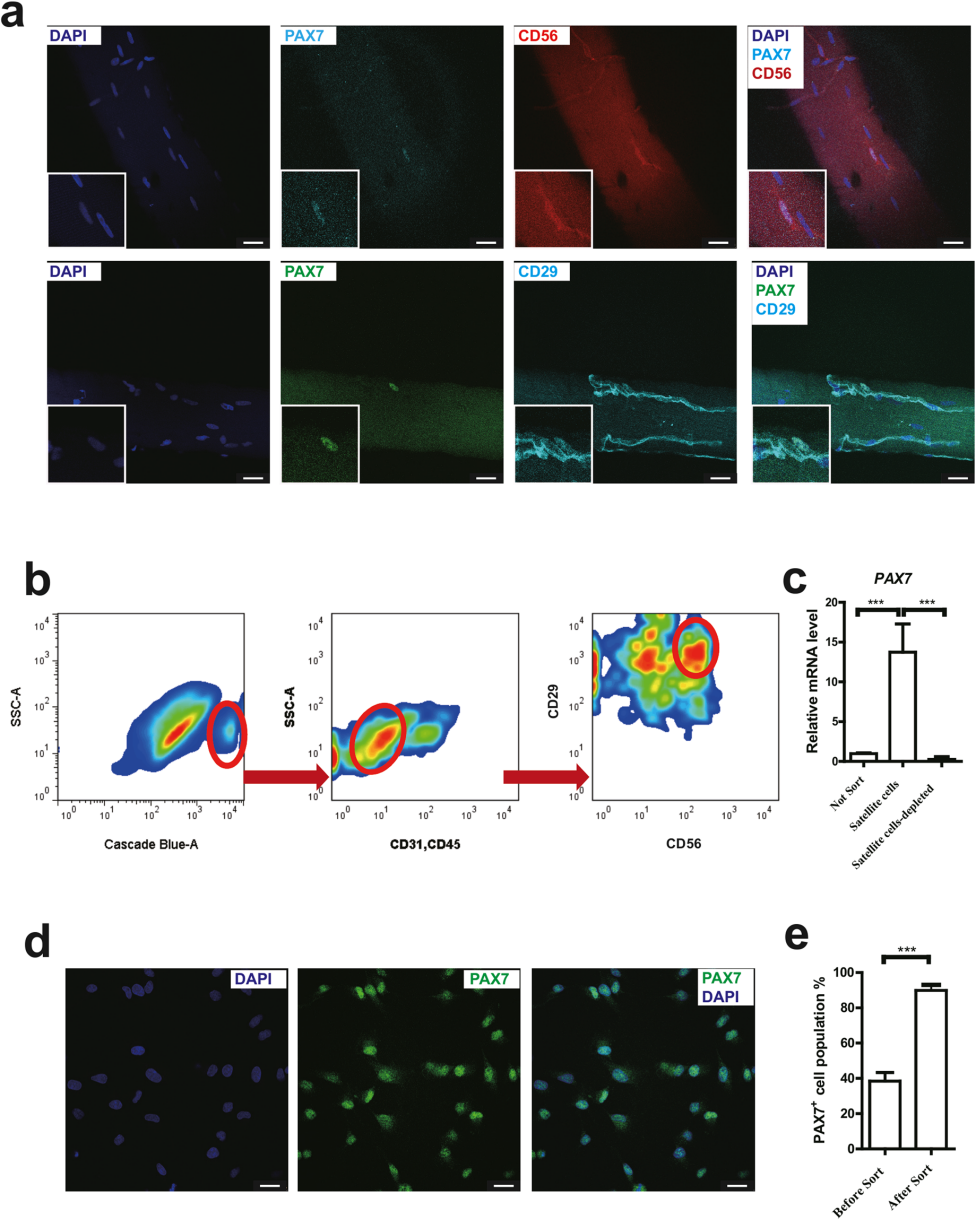
(**a**) Representative Immunofluorescent staining of DAPI, Pax7, CD56 (**a**, top), CD29 (**a**, bottom) on an adult bovine muscle fibre. Scale bars: 25 μm. (**b**) FACS analysis of mononuclear cells from biceps femoris obtained from adult cow. Cells were gated by forward scatter and side scatter (not shown) prior to gating for Hoechst, CD45/31, CD56/29. Red gates (ovals) indicate sub-populations containing bovine satellite cells. (**c**) qRT-PCR analysis of *PAX7* mRNA levels in total cells (not sorted), sorted satellite cells and satellite cells depleted populations after a period of 4 days in culture. (n = 3). (**d**) Representative Immunofluorescent staining of PAX7 in sorted bovine satellite cells cultured for 4 days. Scale bars: 25 μm. (**e**) Quantification of PAX7 immunofluorescent staining of sorted CD56^+^CD29^+^ cells and unsorted cells cultured for 4 days (n = 3). Data are represented as mean ± SEM. Significance was analyzed by Student’s t-test for 2 groups, One-way ANOVA with Bonferroni’s Multiple Comparison Test for more than 2 groups. Asterisks: ***indicates *P* < 0.001.

To isolate bovine satellite cells by FACS method, we firstly analyzed PAX7, CD56 and CD29 protein expression in mature bovine muscle fibers (Fig. 1a). PAX7 is the most specific marker of satellite cells^26,27^ and bovine satellite cells were recognized by PAX7 nuclear staining. Both CD56 and CD29 co-stained with PAX7 in bovine skeletal muscle fibers (Fig. 1a). This suggests that these two proteins might serve as positive markers for bovine satellite cells in FACS method. CD31 and CD45, two antibodies against endothelial cells and hematopoietic cells were utilized as negative markers for sorting satellite cells^16–18^. After isolating the mononuclear cell population obtained from bovine skeletal muscles, Hoechst was used to distinguish cells and tissue debris (Fig. 1b, left). Then the CD31^−^CD45^−^CD56^+^CD29^+^ cells were isolated as bovine satellite cells (Fig. 1b middle, right). *PAX7, PAX3 and MYF5* mRNA was highly expressed in sorted satellite cells compared with unsorted cells or satellite cell depleted populations (Fig. 1c, Supplementary Fig. S1a,b). Immunofluorescent staining for PAX7 further confirmed the satellite cell identity (Fig. 1d). About 92% sorted satellite cells were co-stained with DAPI and PAX7 protein (Fig. 1d,e). We also checked other surface markers and transcriptional factors of sorted satellite cells (Supplementary Fig. S1c–g), such as MYOD^28^ (98.48 ± 1.81%), DESMIN^29^ (98.57 ± 2.13%), M-Cadherin^28,30^ (97.64 ± 0.89%), MYF5^16^ (97.01 ± 2.73%), ITGA7^16^ (98.27 ± 3.03%). These results indicate that bovine satellite cells can be isolated from muscles tissue and purified by FACS sorting using CD31 and CD45 negative selection followed by CD56 and CD29 positive selection.

### p38 inhibition maintains PAX7 expression in bovine satellite cells

**Figure 2:**
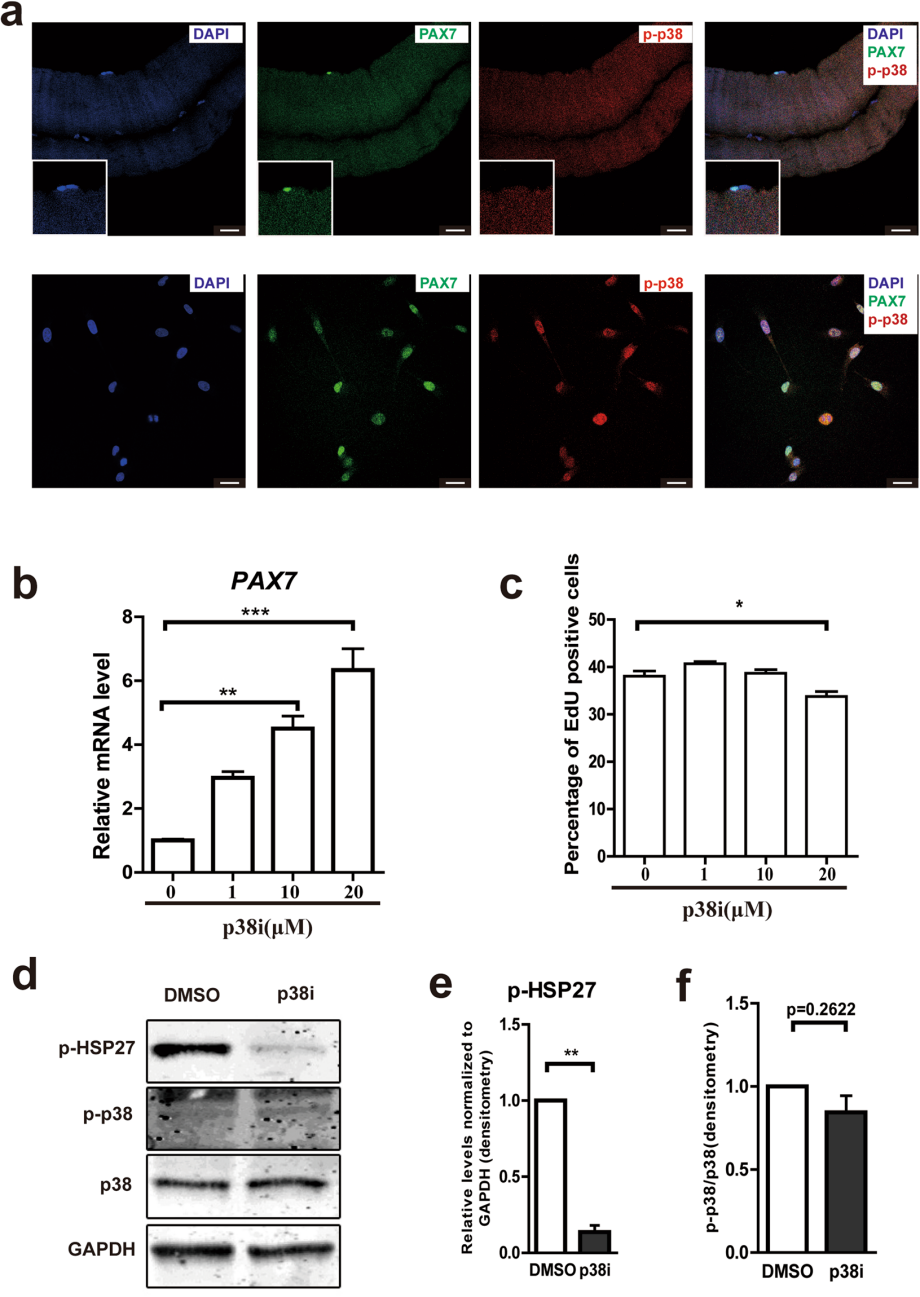
(**a**) Representative immunofluorescent staining of DAPI, PAX7 and p-p38 in quiescent bovine satellite cells (freshly isolated fiber, top) and activated satellite cells in culture (bottom). Scale bars: 25 μm. (**b**) qRT-PCR: concentration-dependent inhibition of PAX7 expression in 2 days cultured satellite cells (passage 2) by p38i inhibitor SB203580. (n = 3). (**c**) Relative percentage of 1.5 hr window EdU incorporation at given concentrations of p38i for satellite cells at passage 2 after 2 days culturing. (n = 4). (**d**) Representative images of immunoblotting against p-HSP27, p-p38, p38, and GAPDH from cell lysates of cells cultured 4 days to 90% confluent in the presence or absence of 10 μM p38i. Full-length blots are presented in Supplementary Fig. S5. (**e**) Relative levels of p-HSP27 normalized to GAPDH are indicated from (**d**). (n = 3). (**f**) Relative levels of p-p38 normalized to p38 from (**d**). (n = 3). Data are represented as mean ± SEM. Significance was analyzed by Student’s t-test for 2 groups, One-way ANOVA with Bonferroni’s Multiple Comparison Test for more than 2 groups. Asterisks: *indicates *P* < 0.05, **indicates *P* < 0.01, ***indicates *P* < 0.001.

To investigate if phosphorylated (activated) p38 (p-p38) is up-regulated during culturing of bovine satellite cells, we stained the p-p38 together with PAX7 in muscle fiber and cultured bovine satellite cells (Fig. 2a). In muscle fibers, only 4.6% of total PAX7^+^ cells (n = 44) is p-p38^+^ as opposed to cultured satellite cells, where 95.7% of PAX7^+^ cells (n = 140) are p-p38^+^. Phosphorylation-p38 was absent from quiescent satellite cells in bovine muscle fibers (Fig. 2a, top), it is activated during cell culture and expansion (Fig. 2a, bottom). Inhibition of p38 MAPK signaling by SB203580 concentration-dependently increased the expression of *PAX7* mRNA (Fig. 2b), whereas the solvent had no effect. Up to a concentration of 10 µM, the p38 inhibitor had no effect on cell proliferation measured by EdU incorporation (Fig. 2c). Effective p38 inhibition by SB203580 was shown by significantly reduced phosphorylation of the p38 substrate HSP27 (Ser82)^17,24^ (Fig. 2d,e and Supplementary Fig. S2a,b). However, in confluent state, the p-p38/p38 ratio were slightly decreased by p38i treatment (Fig. 2d,e). These results show that p38 MAPK signaling is activated in bovine satellite cells during culture and that short-term inhibition of p38 MAPK signaling maintains the *PAX7* expression suggesting that stemness is preserved.

### P38 inhibition reversibly interferes with myoblast differentiation

**Figure 3:**
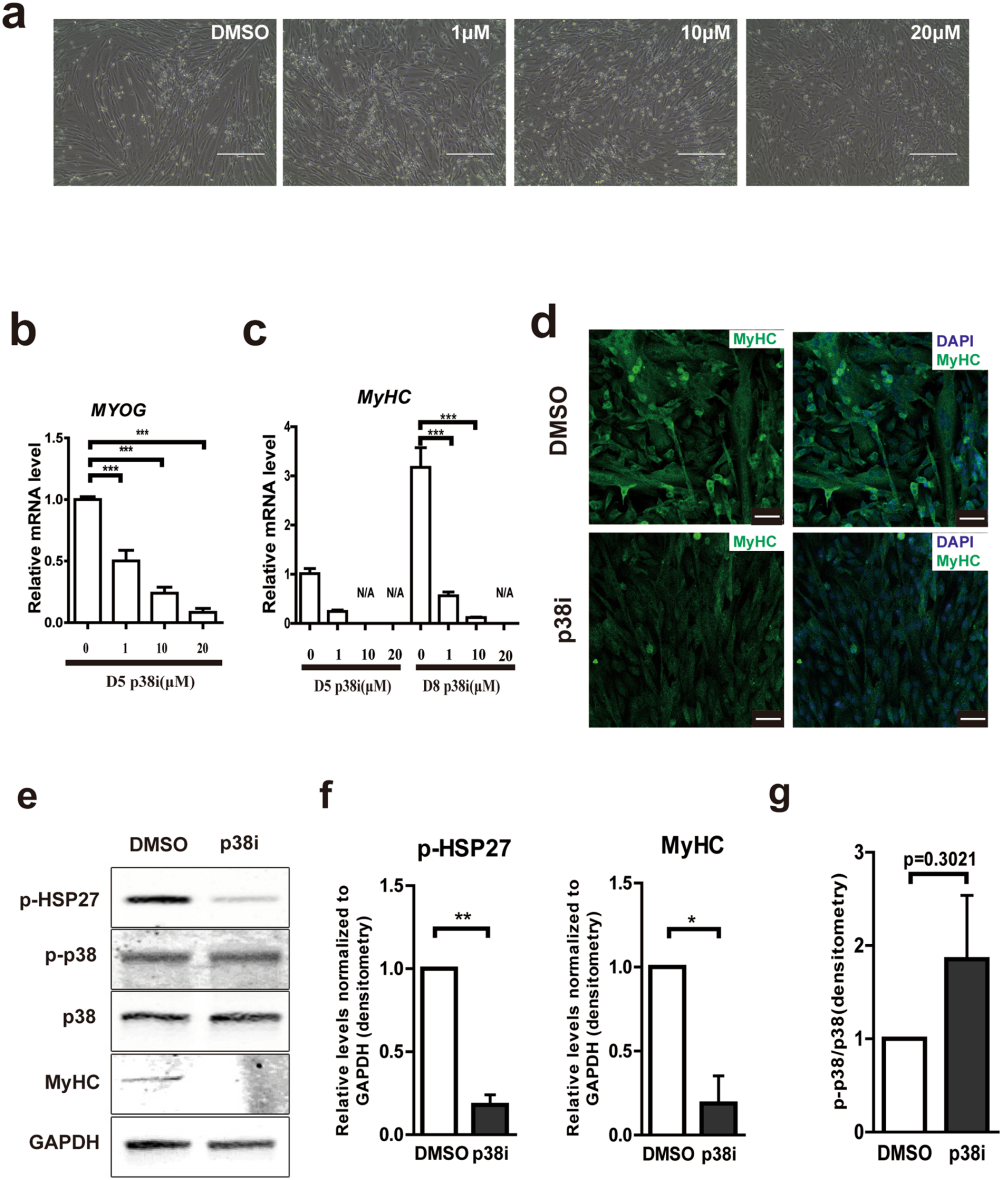
(**a**) Representative phase contrast images of passage 2 bovine satellite cells cultured for 6 days at given concentrations of p38i (SB203580). Scale bars: 200 μm. (**b**) *MYOG* expression after 5 days of culture at given concentrations of p38i. (n = 3). (**c**) *MyHC* expression after 5 and 8 days of culture at given concentrations of p38i. (n = 3). (**d**) Representative immunofluorescent staining of DAPI and MyHC after 6 days of culture in the presence or absence of p38i. Scale bars: 50 μm. (**e**) Representative images of immunoblotting against p-HSP27, p-p38, p38, MyHC and GAPDH from cell lysates of cells cultured 6 days in the presence or absence of 10 μM p38i. Full-length blots are presented in Supplementary Fig. S5. (**f**) Relative levels of p-HSP27 and MyHC normalized to GAPDH are indicated from (**d**). (n = 3). (**g**) Relative levels of p-p38 normalized to p38 from (**d**). (n = 3). Data are represented as mean ± SEM. Significance was analyzed by Student’s t-test for 2 groups, One-way ANOVA with Bonferroni’s Multiple Comparison Test for more than 2 groups. Asterisks: *indicates *P* < 0.05, **indicates *P* < 0.01, ***indicates *P* < 0.001. N/A: not available.

In order to establish p38i effects on the ability of bovine myoblasts to differentiate into myocytes, we cultured bovine satellite cells with different concentrations of the p38 inhibitor. Similar to human^17^, bovine satellite cells automatically fused to form myotubes upon reaching confluency (Fig. 3a, left top, 3d). However, myotube formation was inhibited in p38i treatment groups (Fig. 3a,d). At 10 μM and 20 μM, SB203580 strongly inhibited myotube formation (Fig. 3a,d). *MYOGENIN (MYOG)* and *Myosin Heavy Chain* (*MyHC)*, both markers of myocyte differentiation, were also inhibited by p38 inhibition in a concentration-dependent manner (Fig. 3b,c). Proliferative potential of bovine satellite cells as undifferentiated cells was maintained in p38i treatment group (Supplementary Fig. S3a,b). Immunofluorescent staining for PAX7 showed that the percentage of PAX7 positive cells was also higher in p38i treatment group (Supplementary Fig. S3a,b). Cell lysates of 6 days cultured satellite cells accumulate more p-HSP27, MyHC proteins in the control group than in p38i treatment group (Fig. 3e–g). However, the p-p38/p38 ratio showed a slightly increase. To test whether the inhibition was reversible, we cultured cells with 10 µM p38i for 4 days until confluency and then induced differentiation with differentiation medium in the absence of p38i. Prior p38i treated satellite cells fused into myotubes to the same degree as in the control group (Supplementary Fig. S3e) suggesting that the effect on differentiation is reversible. Taken together, p38i reversibly inhibits bovine satellite cells differentiation in a concentration-dependent way.

### Long-term p38 inhibition delays loss in stemness of bovine satellite cells

**Figure 4:**
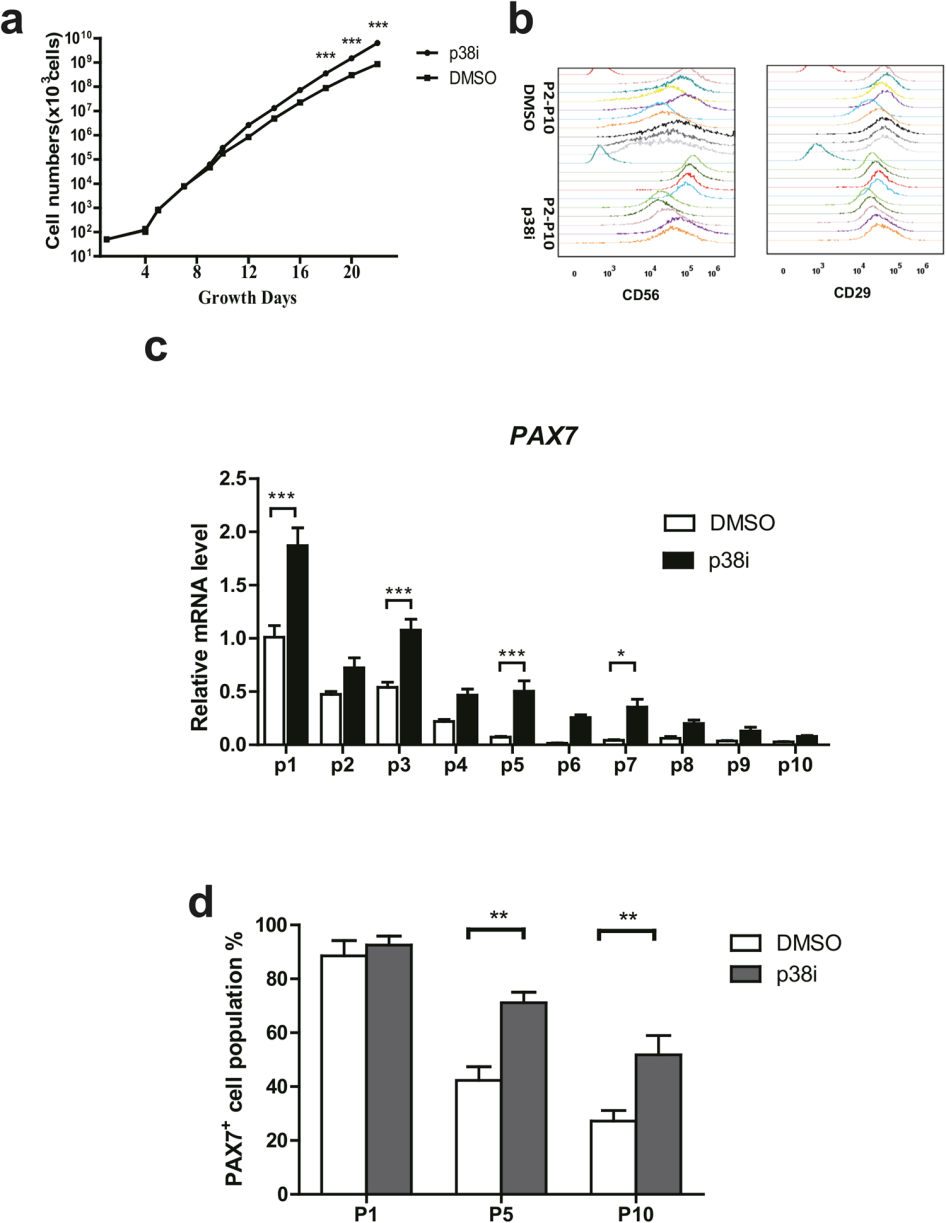
(**a**) Growth curves of bovine satellite cells in absence and presence of p38i. (n = 3). (**b**) FACS analysis of CD56 (left) and CD29 (right) expression from different passages (P2-P10) of bovine satellite cells with DMSO (top 9 lines) and p38i treatment (bottom 9 lines). (**c**) *PAX7* mRNA levels at different passages (n = 3). (**d**) Quantification of PAX7 immunofluorescent staining of different passages. (n = 3). Data are represented as mean ± SEM. Significance was analyzed by 2-way ANOVA and Bonferroni post-tests were used to compare treatment in different passages. Asterisks: *indicates *P* < 0.05, **indicated *P* < 0.01. ***indicates *P* < 0.001.

**Figure 5:**
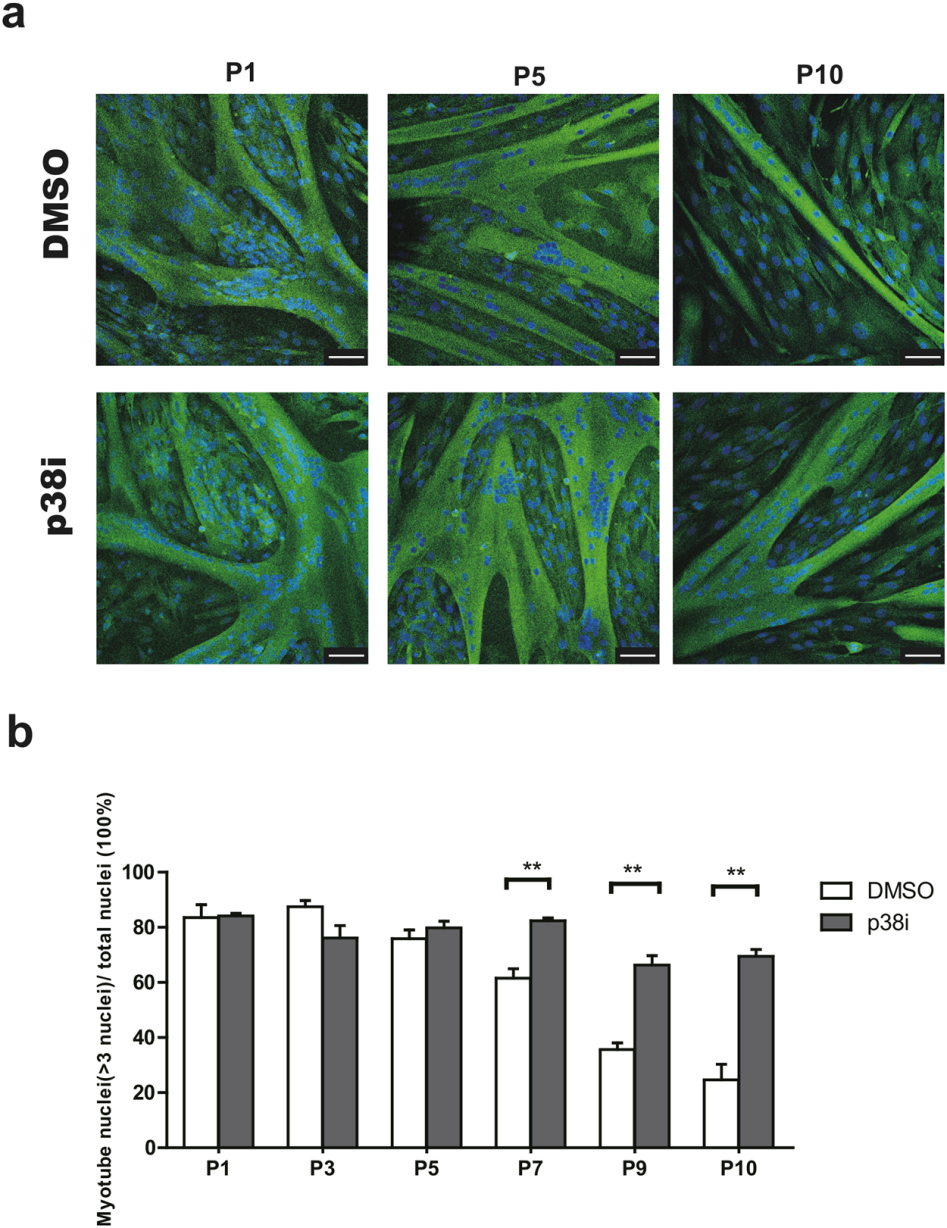
(**a**) Representative immunofluorescent images (Myosin Heavy Chain staining) of myotubes differentiated from P1, P5 and P10 satellite cells. Green indicates MYHC; blue indicates DAPI (nuclei). Scale bars, 75 μm. (**g**) Percentage of nuclei in myotubes differentiated from satellite cells depending of passage (P1, P3, P5, P7, P9, P10). (n = 3). Data are represented as mean ± SEM. Significance was analyzed by 2-way ANOVA and Bonferroni post-tests were used to compare treatment in different passages. Asterisks: **indicates *P* < 0.01.

As p38i can short term help maintain the bovine satellite cells stemness in an undifferentiated state, we next checked the long-term effects of p38i on cultured bovine satellite cells. Firstly, we checked the protein expression of p-HSP27, p-p38, p38 in different passage cells. During long-term culturing *in vitro*, bovine satellite cells showed an up-regulated p-HSP27 expression (Supplementary Fig. S4a). Cells could be propagated logarithmically while long-term exposure to p38 inhibition helped bovine satellite cell proliferation (Fig. 4a, 2-way ANOVA with time and treatment as independent variables: *P* < 0.0001 for time effect, *P* < 0.0001 for p38i treatment effect, *P* < 0.0001 for interaction effect). CD56 and CD29 expression were also analyzed by FACS. Inhibition of p38 maintained CD56 expression compared with control group (Fig. 4b). The expression of *PAX7* mRNA decreased in both groups during passages (Fig. 4c). However, p38i resulted in higher *PAX7* expression levels compared to controls (Fig. 4c, 2-way ANOVA with passage and treatment as independent variables: *P* < 0.0001 for passage effect, *P* < 0.0001 for p38i treatment effect, *P* < 0.0001 for interaction effect). We further stained the PAX7 positivity of cells in each passage (Supplementary Fig. S4b). Consistently, the percentage of PAX7^+^ cells also decreased during passages whereas p38 inhibition continued to show a larger population of PAX7-positive cells (Fig. 4d, 2-way ANOVA with passage and treatment as independent variables: *P* < 0.0001 for passage effect, *P* < 0.0001 for p38i treatment effect, *P* = 0.0469 for interaction effect). To analyze the differentiation ability of myoblasts upon passaging, we induced differentiation when cells were 90% confluence at each passage. After 10 passages, cultured myoblasts lost their capacity to differentiate into myocytes, whereas myoblasts exposed to p38 inhibition maintained this capacity (Fig. 5a,b, 2-way ANOVA with passage and treatment as independent variables: *P* < 0.0001 for passage effect, *P* < 0.0001 for p38i treatment effect, *P* < 0.0001 for interaction effect). Taking together, long-term culture of bovine satellite cells with p38 inhibitor can help maintain the stemness as well as their ability to differentiate.

## Discussion

Maintenance of stemness in a stem cell population is of considerable scientific and societal importance. Recently tissue engineering endeavors, mostly for medical, but even more so for food applications, rely on high expansion numbers of undifferentiated cells that at the same time retain their differentiation capacity^17,19,31–33^. A prerequisite for maintaining stemness is to start with a highly purified stem cell population, since most other cells in culture grow faster than the stem cells, so that the latter will eventually be outnumbered^17,18,34,35^. Even in a highly purified stem cell population, the more differentiated off-spring from asymmetric division, such as myoblasts from satellite cells, will overgrow the stem cells^17,18,36^.

Here, we firstly improved the purity of bovine satellite cells by FACS and established that a combination of negative selection for CD31 and CD45 with a positive selection for CD29 and CD56 results in a high purity satellite cells population as judged by their degree of PAX7 positivity. In this highly purified population, we observed that inhibition of p38 phosphorylation kept the stem cell profile of bovine satellite cells for more passages, while they retained the ability to differentiate into myocytes.

p38α/β MAPKs is required for satellite cells activation and can function as a molecular switch for satellite cell activation even before MyoD up-regulation^37^. Because of serum and growth factor stimulation, p38α/β signaling will continually be activated. Activated p38α/β targets SWI-SNF chromatin-remodeling complex to myogenin loci^38^, which makes the satellite cells generate more differentiated Pax7^−^Myog^+^ daughter cell^24,39,40^. The Myog expression leads to the down-regulation of genes involved in cell cycle progression and promotes differentiation and fusion^41^. This can be seen as a first step towards myoblast differentiation. At the same time, p38α represses Pax7 through interaction between the transcription repressors YY1 and Polycomb Repressive Complex (PRC2)^42,43^, further leading to loss of satellite cell stemness. Modest inhibition of p38α will help maintain PAX7 expression (Fig. 2b)^17,24,40^ and therefore the stemness of satellite cells^44–46^. Total inhibition of p38α/β by SB203580 at 20 µM also inhibited satellite cells proliferation (Fig. 2c) and this is consistent with previous studies^17,37^. At lower concentrations however, and in the presence of FGF-2, p38 inhibition slightly increased proliferation of satellite cells in long-term culture (Fig. 4a), most likely due to maintenance of stemness as PAX7 expression is preserved and *MYOGENIN*, as a marker of differentiation, is decreased. *Myogenin* downregulation by SB203580 has been observed in other studies and in different cell culture models as well^24,38,40^. Together the data show that p38 inhibition keeps satellite cells in an undifferentiated and proliferative state.

Although PAX7 decrease is delayed by p38 inhibition, long-term cultivation of bovine satellite cells still leads to loss of PAX7 expression and also loss of differentiation abilities. (Figs 4, 5). Other interventions may be required to further improve the replicative capacity of satellite cells. In mouse muscle stem cells for instance, the stiffness of the substrate elasticity helped maintaining the stemness of muscle stem cells^24,47–49^. The suitable stiffness for bovine muscle stem cells still needs to be investigated. Another optimal way for keeping the stemness of bovine satellite cells is to culture the bovine satellite cells in hypoxia conditions^50–52^. Hypoxia (1% oxygen) stimulates the proliferation and differentiation of bovine myoblasts^51^. Similar results have been shown in mouse satellite cells: Hypoxia (1% oxygen) conditions favor quiescence of primary mouse myoblasts by upregulating Pax7^52^.

We noticed progressive loss of stemness (PAX7 positivity) during passaging, but also loss of differentiation capacity. Loss of differentiation capacity into myocytes suggests de-differentiation of satellite cells that progresses further than just losing stemness. It is currently unknown what is responsible for this de-differentiation. One option is that the cells transdifferentiate into another lineage. For instance, satellite cells can generate brown fat cells during long-term cultivation^53–55^. The Prdm16 gene controls a brown fat and skeletal muscle switch^53,54^. Whether this switch or other factors are responsible for the general decrease in differentiation capacity of satellite cells and myoblasts in culture, requires further studies.

We conclude that FACS purification of bovine satellite cells using CD29 and CD56 as positive markers increase the stem cell population and that in long-term culture p38 inhibition preserves stemness, proliferation and differentiation of bovine muscle precursors. This might bring large scale bovine muscle cell culture for cultured beef applications closer to reality.

## Materials and Methods

### Bovine muscle tissues

The bovine satellite cells in this study were derived from fresh (within 30 min of euthanasia) muscle samples obtained at a local slaughterhouse.

### Bovine satellite cells isolation

Bovine satellite cells were isolated from 1–2 year old male cattle as previously described and adapted to bovine tissues^15,17,18^. Briefly, freshly harvest bovine muscle was immediately transferred to the lab on ice and washed with 75% ethanol for 1 min, followed by PBS for 2 times. Then, the tissues were mechanically dissected and dissociated with collagenase II (Worthington, CLS-2, 0.2%) in DMEM (Invitrogen, Cat# 41966-29) supplemented with 1% penicillin-streptomycin (P.S. Lonza, Cat# 17-745E) at 37 °C for 1.5 h. The mixture was mixed by vortexing or triturated with pipette once per 10 min. After digestion, 20% FBS in DMEM was added and mixed well with pipette. The muscle fragments were centrifuged at 80 g for 3 min and the supernatant was collected as mononuclear cell suspension. The precipitated debris was again triturated with a 20-gauge needle in PBS and centrifugated at 80 g for 3 min. The supernatant was collected again and mixed with former mononuclear cell suspensions. After centrifugation at 1,000 g for 5 min, the cells were washed twice with PBS followed by DMEM with 20% FBS. After that, the cells were filtered through a 100 µm cell strainer followed by a 40 µm cell strainer. The cells were then centrifuged at 1,000 g for 5 min at 4 °C and incubated with the erythrocyte lysis buffer (ACK) buffer for 5 min on ice. Then the cells were washed twice with PBS and cell pellet was reconstituted with FACS buffer (1% BSA in PBS) or frozen in FBS supplement with 10% dimethyl sulphoxide (DMSO, sigma).

The frozen cells were first recovered in a 37 °C water bath and washed with PBS twice. The cells were resuspended in FACS buffer and stained with APC anti-human CD29 Antibody (1:10, BioLegend, Cat# 303008), PE-Cy^TM^7 anti-human CD56 (1:10, BD, Cat# 335826), FITC anti-sheep CD31 (1:10, BIO-RAD, Cat# MCA1097F), FITC anti-sheep CD45 (1:10, BIO-RAD, Cat# MCA2220F) for 30–45 min on ice. After antibody incubation, the cells were washed twice with cold PBS and reconstituted in F-10 with 20% FBS. The viable CD31^−^CD45^−^CD56^+^CD29^+^ cells were isolated by cell sorting. The negative cells were also isolated to extract RNA. Cell sorting was performed with a BD FACSAria cell sorter using 405, 488 nm and 640 nm lasers. Unstained cells were routinely used to define FACS gating parameters.

### Satellite cell culture and differentiation

Dishes (Corning) were coated with 0.05% bovine collagen type I (Sigma, Cat# C4243). FACS isolated bovine satellite cells and unsorted cells were cultured on collagen-coated dishes in F10 medium (Gibco, Cat# 31550-023) containing 20% fetal bovine serum (FBS, Gibco, Cat# 10500-06), 5 ng/mL bFGF (R&D, Cat# 233-FB-025) and 1% P.S. Where indicated, medium was supplemented with p38i (SB203580, Selleck, Cat# S1076) and DMSO (Sigma, Cat# D8418). For serial expansion, cells were passaged to maintain a density of <60% confluence and counted at each passage. Bovine satellite cells differentiation was induced at 90% confluency with DMEM (Invitrogen, Cat# 41966-29) with 2% FBS. The expanded and differentiated cells were fixed with 4% PFA for immunofluorescent staining. The bright field images were acquired by AMG-EVOS microscope.

### Immunofluorescent analysis of cultured cells

Cultured satellite cells were fixed with ice-cold 4% PFA (in PBS) for 20 min, rinsed with PBS, and permeabilized in 0.5% Triton X-100 (in PBS) for 15 min. Permeabilized cells were blocked and incubated with primary antibodies in 1% BSA (in PBS) overnight at 4 °C. Primary antibodies recognizing mouse Pax7 (1:10, Developmental Studies Hybridoma Bank, Cat# PAX7), mouse myosin heavy chain (1:100, Sigma, Cat# M4276), mouse MyoD (1:200, ABclonal, CAT# A0671), mouse Desmin (1:100, sigma, Cat# D1033), mouse M-cadherin (1:200, BD, Cat# 611100), Rabbit Myf5 (1:50, Santa Cruz, Cat# SC-302), Rabbit ITGA7 (1:100, LifeSpan BioSciences, Cat# LS-C313325), Rabbit p-p38 (1:100, Cell Signaling, Cat#9211). After washing with PBS, cells were incubated with Alexa 488 labeled anti-mouse (1:600, Invitrogen, Cat #A-11001) antibodies for 1 h at room temperature and mounted with VECTASHIELD mounting medium with DAPI (Vector Laboratories Cat# H-1500).

EdU detection was carried-out using a Click-It EdU detection kit (Life Technologies, Cat# C10337) according to the manufacturer’s instructions.

For the FACS analysis of cultured satellite cells, about 10^5^ cells were collected at each passage. The cells were fixed with 4% PFA for 15 min and washed two times for 5 min with PBS. The cells were then incubated with Alexa Fluor 488 anti-human CD29 (1:10, BioLegend, Cat#303016), PE-conjugated anti-human CD56 (1:10, BioLegend, Cat# 304606) in 1% BSA in PBS for 30–45 min. After antibody incubation, the cells were washed with PBS for two times and reconstituted in PBS. FACS analysis was performed with the Cytomics FC500 Flow Cytometer (Beckman Coulter) cell analyzer. Unstained cells were routinely used to define FACS gating parameters.

### Western blots analysis of cultured cells

Western blots were either performed from total cell lysates obtained by lysing cells directly with RIPA buffer complemented with PMSF, protease inhibitor cocktail and sodium orthovanadate (Santa Cruz, CAT# sc-24948). Proteins concentration was determined using BCA protein assay kit (Thermo). SDS-PAGE electrophoresis was carried out in 7.5% pre-cast polyacrylamide gels (Bio-Rad, CAT# 5671023). After transfer onto nitrocellulose, membranes were blocked for 30 min with Odyssey Blocking Buffer in PBS (Part Number: 927-40000, LI-COR Biosciences) and probed overnight with primary antibody: p-HSP27(Ser 82) (1:500, Cell Signaling, CAT# 9709), p-p38 (1:1000, Cell Signaling, CAT# 9211), p38 (1:1000, Cell Signaling, CAT# 8690), MyHC (1:500, Millipore, CAT# 05-716), GAPDH (1:500, Millipore, CAT# MAB374). After that, IRDye700-conjugated or IRDye800-conjugated secondary antibodies were used and visualized with the Odyssey infrared detector (LI-COR Biosciences, Westburg, Leusden, The Netherlands). For Protein quantification a, we used the background correction option in the software of the supplier (Image Studio™ Software for the Odyssey CLx-LI-COR Biosciences) and scanned the corresponding band of the protein of interest.

### Bovine muscle fiber isolation and analysis

The bovine muscle fiber was isolated as previously described and adapted to bovine tissues^15^. After procurement, part of the specimen was immediately fixed in 4% paraformaldehyde (PFA) at room temperature for 20 min and washed with PBS. The sample was then embedded in 30% glycerol in PBS overnight at 4 °C, then 50% glycerol in PBS overnight at 4 °C, then 80% glycerol in PBS overnight at 4 °C and finally 100% glycerol. The fibers were stored in 100% glycerol at 4 °C until dissection.

Single fibers were dissected using fine forceps under a dissecting microscope. Single fibers were washed in PBS for 15 min at room temperature, then permeabilized with 0.5% Triton X-100 (Sigma-Aldrich, Cat#X-100) for 10 min, and washed twice with PBS for 8 min. Single fibers were then blocked with 3% goat serum in PBS for 1 h at room temperature and incubated overnight at 4 °C with the following primary antibodies: mouse monoclonal anti-PAX7 (1:10 Developmental Studies Hybridoma Bank, Cat#PAX7), p-p38 (1:100, Cell Signaling, Cat#9211), The next day, after PBS wash at room temperature for 15 min, fibers were incubated with Alexa 488 labeled anti-mouse (1:600, Invitrogen, Cat#A-11001), Alexa 594 labeled anti-rabbit (1:600, Invitrogen, Cat#:A-21207) antibodies for 1 h at room temperature. Then mounted with VECTASHIELD mounting medium with DAPI (Vector Laboratories Cat# H-1500). All images were acquired by Leica SP6 confocal microscope and processed with Adobe Photoshop CS5 to adjust brightness and contrast for publication.

For fibers co-stained with PAX7 and CD56/CD29, PAX7 staining was firstly performed. After incubation with the secondary antibody (Alexa 488 labeled anti-mouse for Pax7 and CD29 co-staining, Alexa 647 labeled anti-mouse (1:600, Invitrogen, Cat#:A-31571) for PAX7 and CD56 co-staining) above, sections were washed twice with PBS for 8 min and incubated with the APC anti-human CD29 (1:10, BioLegend, Cat# 303016) or PE anti-human CD56 (1:10, BioLegend, Cat# 304606) antibody for 2 h at room temperature. After washing with PBS for 3 times, fibers were mounted with VECTASHIELD mounting medium with DAPI (Vector Laboratories, Cat# H-1500). All images were acquired by Leica SP6 confocal microscope and processed with Adobe Photoshop CS5 to adjust brightness and contrast for publication.

### Gene expression analysis

RNA was extracted from cells using the RNeasy Micro Kit (QIAGEN, Cat#74004) including RNase-Free DNase Set (QIAGEN, Cat# 79254) according to the manufacturer’s instruction. 500 ng of total RNA from each sample was reverse transcribed to cDNA using iScript cDNA Synthesis Kit (Bio-Rad, Cat#1708891) according to the manufacturer’s instruction. Relative gene expression was performed in triplicate using a SYBR Green PCR master mix on an CFX 96 Real Time PCR system (Bio-Rad).

The primers used in these assays were the followings:

PAX7-F, 5′-CTCCCTCTGAAGCGTAAGCA-3′, PAX7-R, 5′-GGGTAGTGGGTCCTCTCGAA-3′;

PAX3-F, 5′-CAAAGCTTACAGAGGCCCGA-3′, PAX3-R, 5′-GGTCTCTGACAGCTGGTACG-3′

MYF5-F, 5′-TCTATCTCTCTGCTGTCCAGGC-3′, MYF5-R, 5′-AACTCGTCCCCGAACTCAC-3′;

MYOG-F, 5′-GCGCAGACTCAAGAAGGTGA-3′, MYOG-R, 5′-TGCAGGCGCTCTATGTACTG-3′;

MyHC-F, 5′-AGAGCAGCAAGTGGATGACCTTGA-3′,

MyHC-R,5′-TGGACTCTTGGGCCAACTTGAGAT-3′;

GAPDH-F, 5′-CACCCTCAAGATTGTCAGC-3′, GAPDH-R, 5′-TAAGTCCCTCCACGATGC-3′.

### PAX7^+^ cells population, differentiation efficiency, EdU and pHSP27 percentage measurement

PAX7^+^ cell populations were expressed as the number of nuclei co-stained with DAPI and PAX7 divided by the total number of nuclei in the same field. More than 300 nuclei from 5 or more randomly chosen fields were analysed.

The percentage of myotube nuclei per condition was expressed as the nuclei inside myotube (at least 3 nuclei) divided by total nuclei in the same field. More than 1000 nuclei from 4 randomly chosen fields were analysed.

For EdU detection, cells cultured in BD Falcon 96 wells HTS Imaging microplates were imaged using a High-Content Analyzer (BD Pathway 855) with a 4× objective. In total 16 images were taken for each condition and individual cell segmentation and analysis was performed using the BD Attovision software (BD Biosciences, version 1.6). Hoechst positive cells were segmented and counted, then the same was done for EdU positive cells. The numerical data were further analyzed with Kaluza software (version 1.3; Beckman Coulter), where the EdU positive percentage was determined for more than 6000 nuclei from 16 randomly chosen fields.

For pHSP27(Ser82) detection, cells cultured in 96-well plate were imaged using a High-Content Imager (BD Pathway 855) with a 20× objective. Together 16 images were taken for each condition. Using AttoVision-software, the entire cell was segmented by fluorescence intensity and the pHSP27 (Ser82) positive speckles were counted in each cell. The average speckles were defined between different groups. More than 700 nuclei from 16 randomly chosen fields were analysed.

### Statistics

Statistical analyses were performed using GraphPad Prism 5 (GraphPad Software). For comparisons of two treatment groups, a Student’s t-test was used. For more than two groups, One-way ANOVA with Bonferroni’s Multiple Comparison Test was used. For long-term culture experiments, 2-way ANOVA was used to distinguish the p38i treatment and passages effects. Results were means ± S.E.M. unless otherwise stated. *P* < 0.05 was considered significant.

### Data availability

The datasets generated during and/or analysed during the current study are available from the corresponding author on reasonable request.

## Electronic supplementary material

Supplemental Information
