# Supplementary material for: Maintaining bovine satellite cells stemness through p38 pathway
Source: Sci Rep. 2018 Jul 17;8:10808. doi: 10.1038/s41598-018-28746-7 (PMC6050236; doi:10.1038/s41598-018-28746-7)
Supplement: Supplementary file 1 — Supplemental Information [file 41598_2018_28746_MOESM1_ESM.pdf]

Supplemental Information

Maintaining bovine satellite cells stemness through p38 pathway

Authors: Shijie Ding, Swennen GNM, Tobias Messmer, Mick Gagliardi, Daniël G. M. Molin, Chunbao Li,

Guanghong Zhou and Mark J. Post

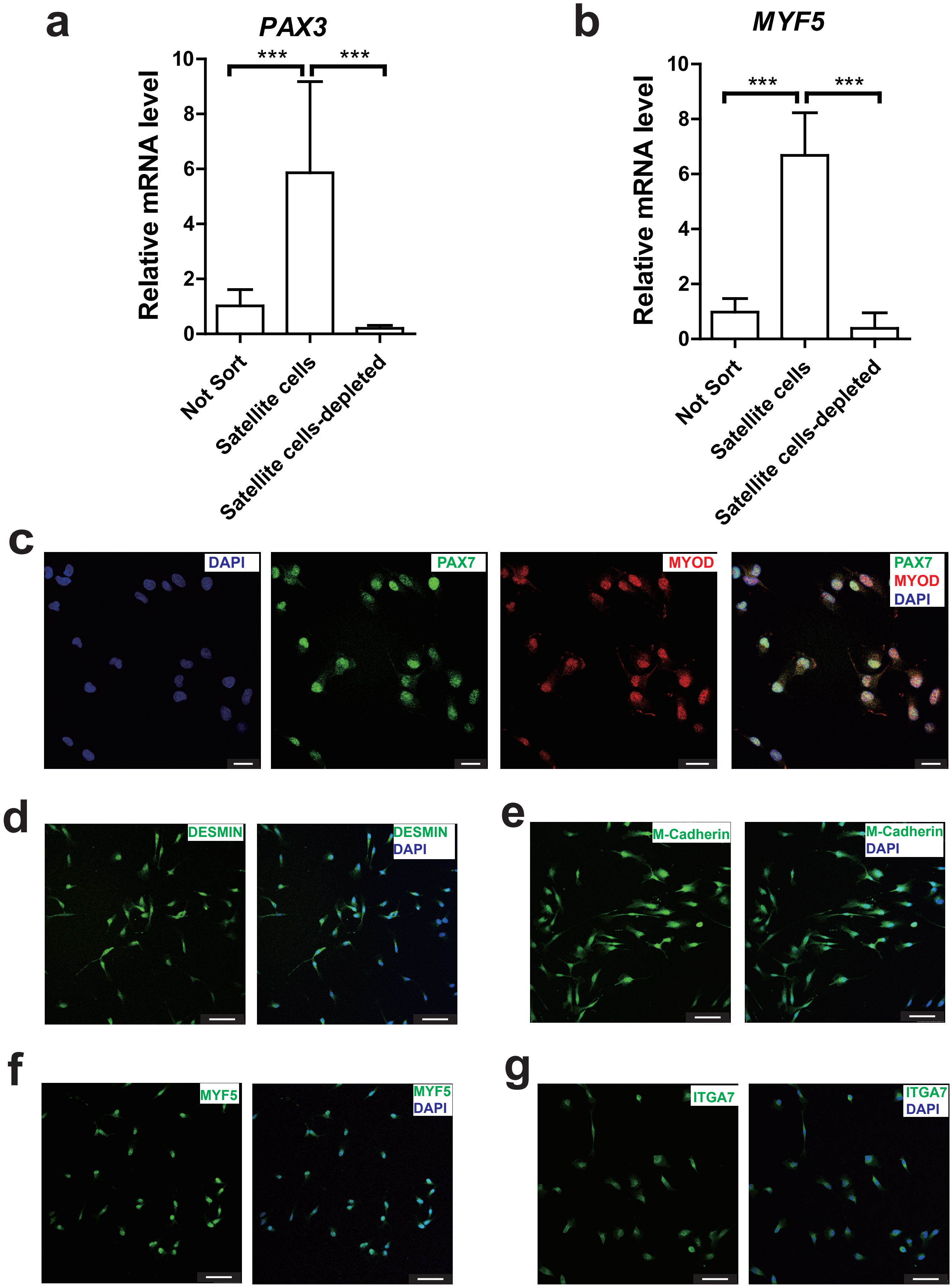

a
PAX3
Relative mRNA level
10
8
6
4
2
0
***
***
Not Sort
Satellite cells
Satellite cells-depleted
b
MYF5
Relative mRNA level
10
8
6
4
2
0
***
***
Not Sort
Satellite cells
Satellite cells-depleted
c
DAPI
PAX7
MYOD
PAX7
MYOD
DAPI
d
DESMIN
DESMIN
DAPI
e
M-Cadherin
M-Cadherin
DAPI
f
MYF5
MYF5
DAPI
g
ITGA7
ITGA7
DAPI

Figure S1. Characterization of sorted bovine satellite cells. Related to Figure 1.

(a) qRT-PCR analysis of *PAX3, MYF5* mRNA levels in total cells (not sorted), sorted satellite cells and satellite cells depleted populations after a period of 4 days in culture (n=3). (b) Immunofluorescent staining of PAX7 and MYOD in sorted bovine satellite cells cultured for 4 days. Scale bars: 25µm. (c) Immunofluorescent staining of DESMIN, M-Cadherin, MYF5, ITGA7 in sorted bovine satellite cells cultured for 4 days. Scale bars: 75µm. Data are represented as mean ± SEM. Significance was analyzed by One-way ANOVA with Bonferroni's Multiple Comparison Test. Asterisks: *** indicates $P < 0.001$.

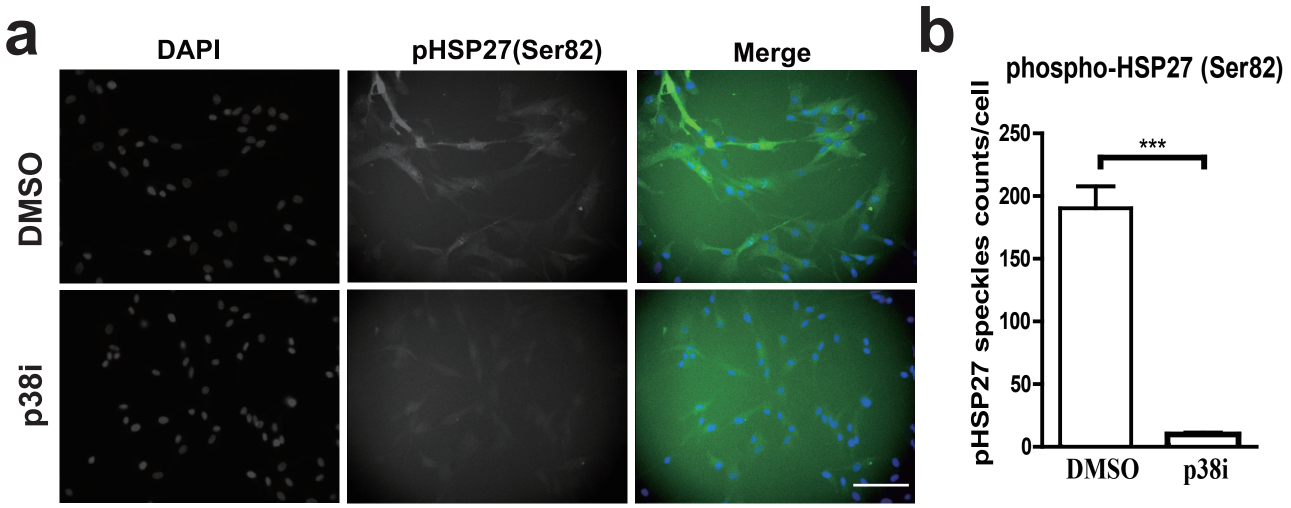

a
DAPI
pHSP27(Ser82)
Merge
DMSO
p38i
b
phospho-HSP27 (Ser82)
pHSP27 speckles counts/cell
250
200
150
100
50
0
***
DMSO
p38i

Figure S2. p38i treatment repressed phosphorylation of the p38 substrate HSP27 (Ser82). Related to Figure 2.

(a) Representative IF analysis of phospho-HSP27 (Ser82) in passage 2 bovine cells cultured for 2 days in the presence or absence of p38i. Scale bar: 100 µm (b) Quantification of IF for phospho-HSP27 (Ser82) per cell from (a). (n=3). Data are represented as mean ± SEM. Significance was analyzed by Student's t-test. Asterisks: *** indicates $P < 0.001$.

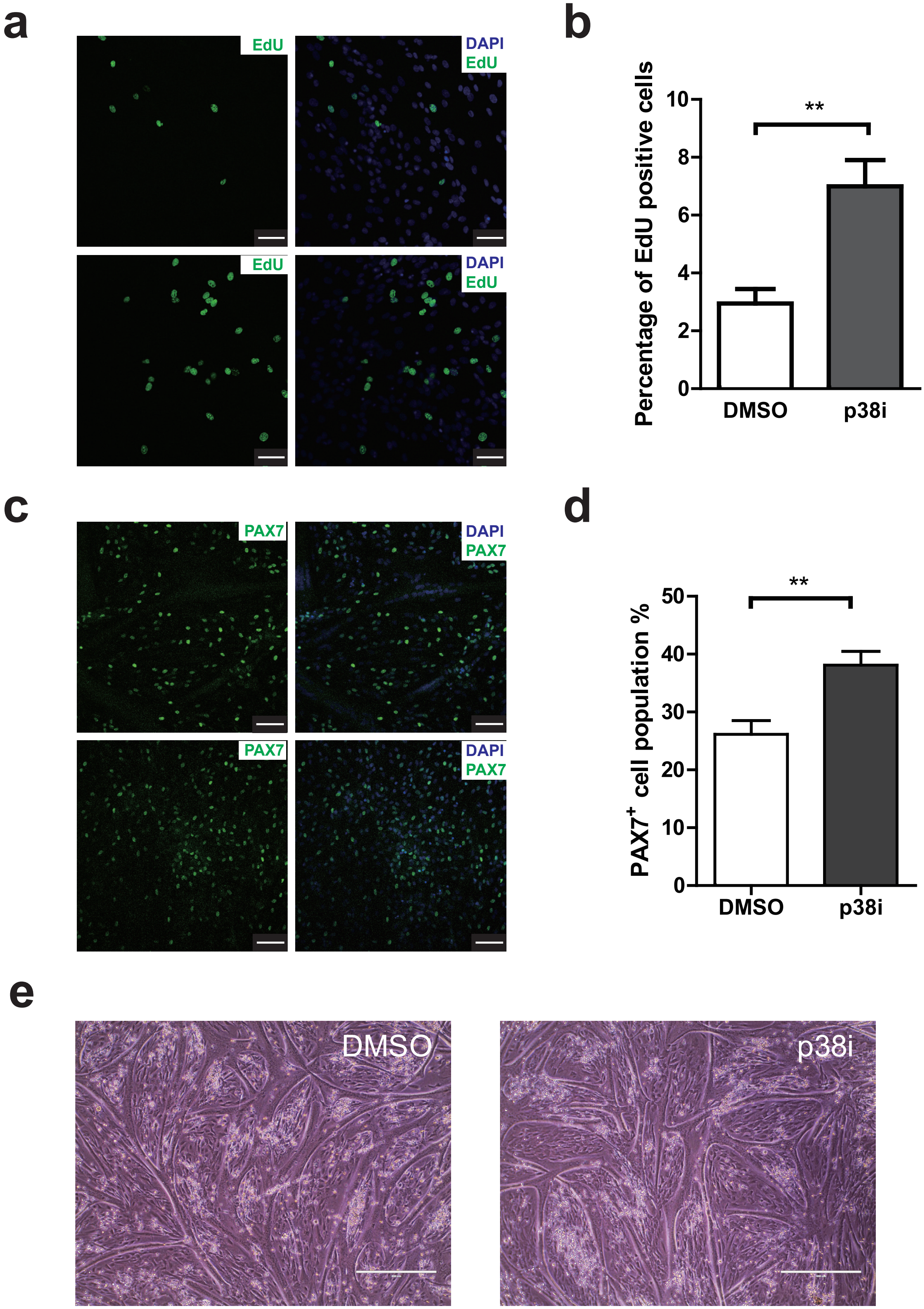
a
EdU
DAPI
EdU
EdU
DAPI
EdU
b
Percentage of EdU positive cells
10
8
6
4
2
0
**
DMSO
p38i
c
PAX7
DAPI
PAX7
PAX7
DAPI
PAX7
d
PAX7+ cell population %
50
40
30
20
10
0
**
DMSO
p38i
e
DMSO
p38i

Figure S3. p38i treatment maintains the proliferation and PAX7 population of satellite cells. Related to Figure 3.

(a) Representative DAPI and EdU immunofluorescent staining of 1 h window EdU incorporation after 6 days of culture in the absence (top panels) or presence (bottom panels) or of p38i. Scale bars: 50µm. (b) Relative percentage of EdU from (a). (c) Representative immunofluorescent staining of DAPI and PAX7 after 6 days of culture in the absence (top panels) or presence (bottom panels) of p38i. Scale bars: 75µm. (d) Quantification of PAX7 immunofluorescent staining from (c). (e) Representative phase contrast images of differentiated bovine satellite cells. Passage 2 satellite cells are originally cultured in F-10 medium with DMSO or 10µM p38i for 4 days before differentiation. Scale bars:400µm. Data are represented as mean ± SEM. Significance was analyzed by Student's t-test. Asterisks: ** indicates $P < 0.01$.

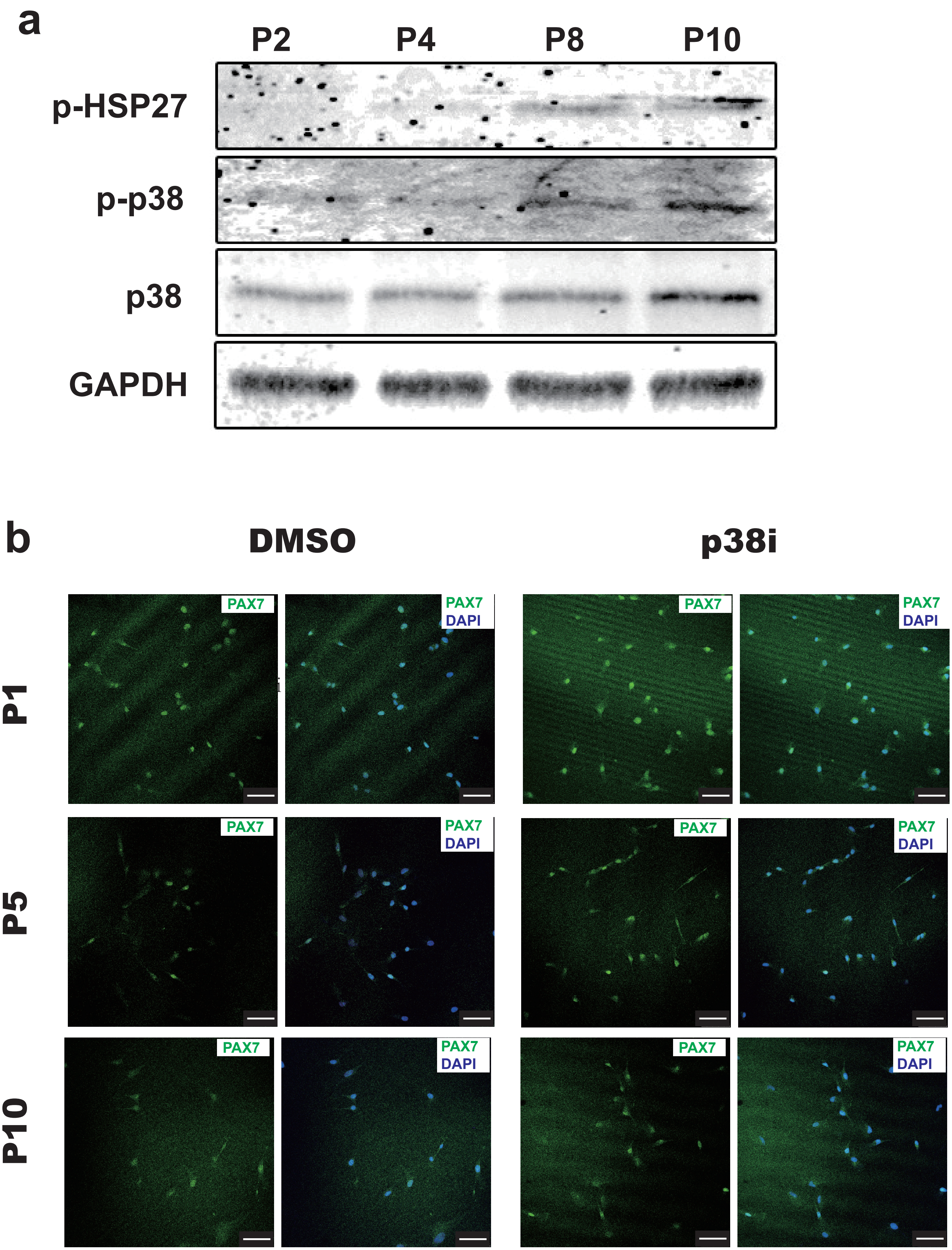
a
P2
P4
P8
P10
p-HSP27
p-p38
p38
GAPDH
b
DMSO
p38i
P1
P5
P10
PAX7
PAX7
DAPI

Figure S4. p38-MAPK signal is up-regulated during long-term culturing and p38i treatment maintains PAX7 population. Related to Figure 4.

(a) Representative images of immunoblotting against p-HSP27, p-p38, p38 and GAPDH of myoblast cells from P2, P4, P8, P10 cultured in F-10 medium. Full-length blots are presented in Supplementary Figure S5.

(b) Representative Immunofluorescent staining of PAX7 in different passage myoblast cells cultured in the presence or absence of p38i. Scale bars: 75µm.

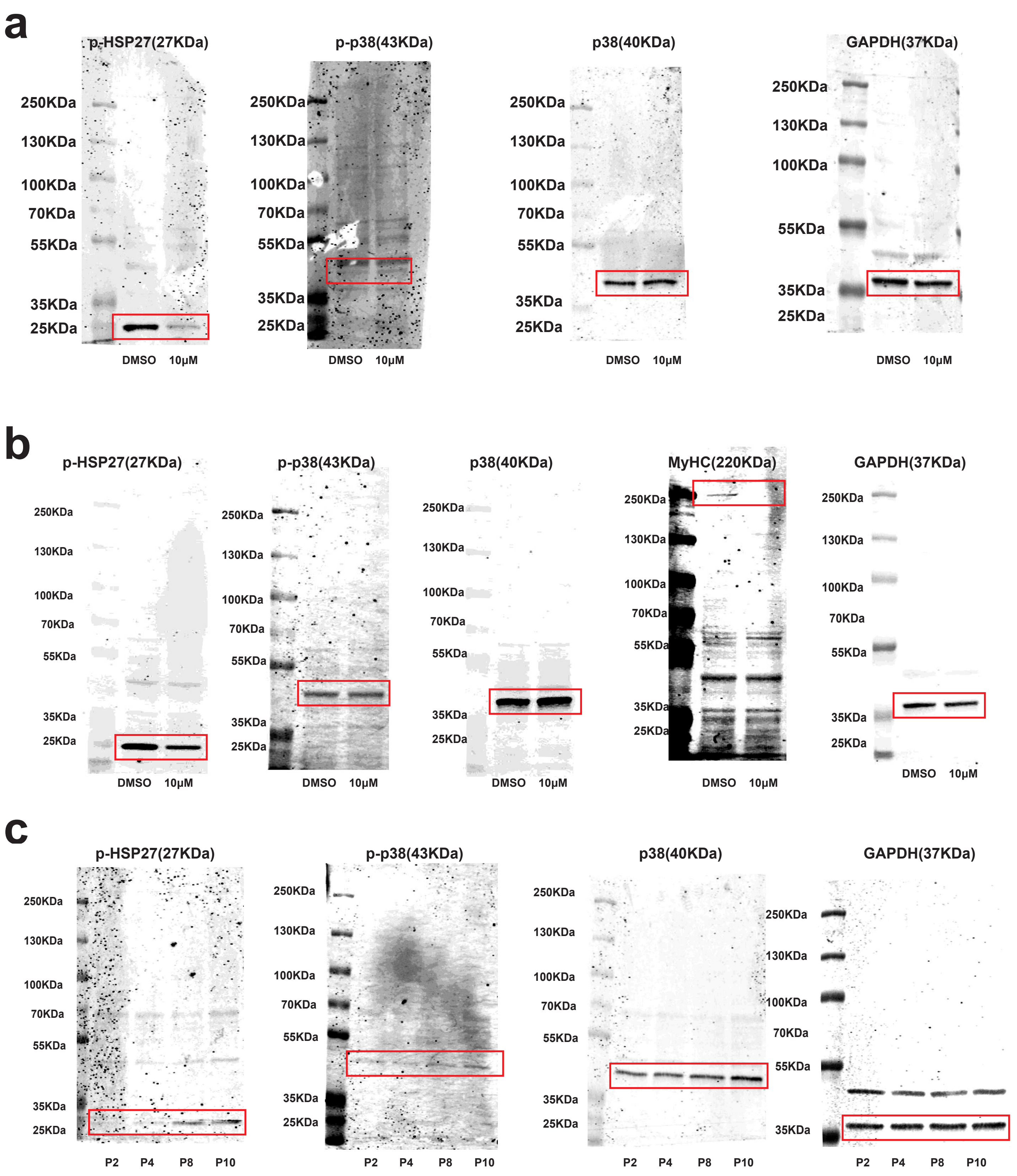
a
p-HSP27(27KDa)
p-p38(43KDa)
p38(40KDa)
GAPDH(37KDa)
250KDa
130KDa
100KDa
70KDa
55KDa
35KDa
25KDa
DMSO 10μM
b
p-HSP27(27KDa)
p-p38(43KDa)
p38(40KDa)
MyHC(220KDa)
GAPDH(37KDa)
DMSO 10μM
c
p-HSP27(27KDa)
p-p38(43KDa)
p38(40KDa)
GAPDH(37KDa)
P2 P4 P8 P10

Figure S5. Full-length western blots. Related to Figure 2, Figure3, Figure S4.

(a) Representative full-length immunoblotting blots against p-HSP27, p-p38, p38 and GAPDH from cell lysates of cells cultured 4 days to 90% confluent in the presence or absence of 10μM p38i. (b) Representative full-length immunoblotting blots against p-HSP27, p-p38, p38, MyHC and GAPDH from cell lysates of cells cultured 6 days in the presence or absence of 10μM p38i. (c) Representative full-length immunoblotting blots against p-HSP27, p-p38, p38 and GAPDH of myoblast cells from P2, P4, P8, P10 cultured in F-10 medium.
